# Surveillance of osteoarticular infections caused by *Staphylococcus aureus* in a paediatric hospital in Mexico City

**DOI:** 10.3389/fcimb.2022.999268

**Published:** 2022-12-08

**Authors:** Nancy Evelyn Aguilar-Gómez, Jocelin Merida-Vieyra, Oscar Daniel Isunza-Alonso, María Gabriela Morales-Pirela, Oscar Colín-Martínez, Enrique Josué Juárez-Benítez, Silvestre García de la Puente, Alejandra Aquino-Andrade

**Affiliations:** ^1^ Department of Paediatric Infectious Diseases, Instituto Nacional de Pediatria, Mexico City, Mexico; ^2^ Molecular Microbiology Laboratory, Instituto Nacional de Pediatria, Mexico City, Mexico; ^3^ Department of Orthopaedic Surgery, Instituto Nacional de Pediatria, Mexico City, Mexico; ^4^ Departament of Research Metodology, Instituto Nacional de Pediatria, Mexico City, Mexico

**Keywords:** *Staphylocoocus aureus*, osteomyelitis, molecular epidemiology, Panton Valentine leukocidin, pediatrics, Mexico

## Abstract

*Staphylococcus aureus* is the main aetiologic agent of osteoarticular infections (OAIs) in paediatric patients. The aim of this prospective unicenter study was to describe the phenotypic and genotypic characteristics of *S. aureus* isolates obtained from OAIs in paediatric patients admitted to tertiary care hospital. Through a surveillance program called *OsteoCode*, a multidisciplinary team was created and we identified 27 patients with OAIs caused by *S. aureus* from 2019 to 2021. The susceptibility profile, virulence factors, biofilm formation, pulsed-field gel electrophoresis (PFGE), clonal complex (CC) and sequence type (ST) were determined. In addition, the clinical characteristics and evolution of the patients presented six months after the diagnosis of OAIs were described. Ninety-two percent of the isolates were methicillin-sensitive *S. aureus* (MSSA). In methicillin-resistant *S. aureus* (MRSA), SCC*mec-*II and SCC*mec-*V were detected. The *pvl* gene was only observed in MSSA (18.5%) and was associated with highest fever (*p*=0.015), multiple localization (*p=*0.017), and soft tissue sites of infection beyond the bone (pyomyositis, pulmonary abscess) (*p=*0.017). Biofilm formation was detected in 55.6% of isolates. The most common CC were CC5 and CC30 which represent the most common linages for bone and joint infections worldwide. The isolates were distributed in different STs, and ST672 was predominant. MRSA were associated with a longer duration of intravenous treatment and a prolonged hospital stay (*p=*0.023). Recurrent infection occurred in five children and orthopaedic complications in 33.3% of patients. This is the first study that reflects the epidemiology of *S. aureus* in OAIs in paediatric patients in Mexico; a clear predominance of MSSA distributed in different STs was observed. Our findings highlight that a multidisciplinary team is required for the diagnosis and treatment of OAIs.

## Introduction

Osteoarticular infections (OAIs) include osteomyelitis (OM), osteoarthritis (OA), septic arthritis (SA) of the native joint and infections associated with prosthetic devices. Early diagnosis, identification of etiologic agent and effective treatment reduce the complications of these conditions and require a multidisciplinary health care team formed by orthopaedists, radiologists, infectologists, microbiologists and rehabilitators.

In developing countries, the incidence of OM among children varies widely from 43 to 200 in 100,000 and for SA from 5 to 20 per 100,000 new cases (Gillespie, 1985; Rossaak and Pitto, 2005; Riise et al., 2008; Arnold and Bradley, 2015; Chiappini et al., 2016; Gigante et al., 2019). In the last 20 years, an increased incidence of this group of diseases has been observed (Ilharreborde, 2015). In 2012, annual increases of 33% and 63% in OM and OA cases, respectively, were reported in the USA (Safdieh et al., 2019). In Mexico, OAIs are not notifiable diseases, so their incidence in the paediatric population is unknown (The Mexican Secretariat of Health, 2021).

*Staphylococcus aureus* is the main etiological agent of OAIs, and its frequency is estimated at 60-75% in OM (Kavanagh et al., 2018; Pimentel de Araujo et al., 2021) and 49% in SA (Nossent et al., 2021). Currently, OM due to *S. aureus* represents a diagnostic and therapeutic challenge, and there is recurrence and persistence of infection in 40% of cases (Kavanagh et al., 2018). Orthopaedic complications in OAIs due to *S. aureus* occur in 9.4% of paediatric patients; the most common is chronic OM, which affects 51.8% of these patients, followed by pathological fracture in 29.6%, growth arrest in 14.8% and avascular necrosis in 7.5% (McNeil et al., 2019).

Identifying the etiological agent of the OAIs is essential for an appropriate antimicrobial treatment. Although microbial culture remains the gold standard for the diagnosis of these infections in 35-85.9% of cases the etiological agent cannot be identified despite an adequate sample of blood, joint fluid or bone (Gornitzky et al., 2020; Trobisch et al., 2022). Molecular tests have recently been developed for the diagnosis of OAIs, being useful in patients who received antibiotics or to identify fastidious microorganisms (Church et al., 2020; Wallander et al., 2022; Lim et al., 2022).However, they have limitations since there may be false positives with contaminating microorganisms of the skin or DNA of non-viable bacteria, do not predict susceptibility profile, and require adequate validation and interpretation (Church et al., 2020; Wallander et al., 2022; Lim et al., 2022). The identification with the sequencing of the 16S rRNA gene is the most used strategy to determine the causal agent, the sensitivity and specificity range from 67.1% to 92.5% and 78.6% to 97.8%, respectively (Jacquier et al., 2019; Yang et al., 2020; Zhang et al., 2020; Lim et al., 2022).

Information about the molecular characteristics of *S. aureus* isolates from OAIs in the paediatric population is limited, and a predominance of methicillin-sensitive *S. aureus* (MSSA) has been observed (58.3-76.9%) (Kechrid et al., 2011; Gaviria-Agudelo et al., 2015; Bouras et al., 2018; Kok et al., 2018; Park et al., 2019). According to the Network for the Research and Surveillance of Drug Resistance, the frequency of methicillin-resistant *S. aureus* (MRSA) in Mexico has decreased from 44.5% in 2009 to 26.2% in 2018. It was more common in respiratory (21.4%), blood (16.7%) and urine (9.1%) samples (Garza-González et al., 2019); however, information about *S. aureus* from bone samples or joint fluid in paediatric patients is limited (Vazquez-Rosas et al., 2021).

The pathogenesis of *S. aureus* in OAIs is related to its ability to produce several virulence factors associated with invasion and persistence in tissues, including adhesins, toxins, exoenzymes, immune evasion factors, and superantigens (Collins et al., 2018; Urish and Cassat, 2020). Among the most important virulence genes for adherence are the microbial surface components recognizing adhesive matrix molecules (MSCRAMMs) such as ClfA, ClfB, FnbA, FnbB, Cna and SpA that, together with other proteins, participate in the early stages of infection, bone tropism, colonization of periprosthetic tissues and biofilm formation (Testoni et al., 2011). Once infection is established, *S. aureus* is persistent due to fibronectin-binding factors A and B (FnbA and FnbB); on the other hand, invasins such as Panton-Valentine leukocidin (PVL) and haemolysin (Hla) damage leukocytes and contribute to the pathogenesis of OM and its complications (Cue et al., 2012; Urish and Cassat, 2020). Staphylococcal enterotoxins and toxic shock syndrome toxin 1 (*tsst-1*) act as superantigens and their role in the pathogenesis in OAIs infections is unclear (Cunningham et al., 1996).

Most of the information on the pathogenesis of *S. aureus* in OAIs comes from studies *in vitro* (Peacock et al., 2000; Elasri et al., 2002; Bocchini et al., 2006; Löffler et al., 2010; Alonzo et al., 2012), in which the expression of different virulence factors has been observed to be associated with invasion and persistence of *S. aureus* in osteoarticular tissue (Testoni et al., 2011). One study reported that the *fnbA, cna, sdrE, sej, eta, hlg* and *ica* genes were the most common in invasive *S. aureus* isolates (Peacock et al., 2002). In another study of paediatric patients with acute haematogenous OM, 40 *S. aureus* genes associated with severity were identified, which indicated that the combination of the expression of these genes could be related to the pathogenesis of the infection (Collins et al., 2018).

OAIs due to PVL-producing *S. aureus* are emerging diseases worldwide; this toxin has been detected in both MSSA and MRSA. Patients infected by these isolates exhibit clinical severity, extraosseous complications (subperiosteal abscess, pyomyositis, necrotizing fasciitis, multifocal disease, and septic emboli) and orthopaedic sequelae in 33 to 85% of cases (Ilharreborde, 2015; Jiang et al., 2017; Moutaouakkil et al., 2022). PVL detection is important to start specific antimicrobial therapy, such as an antistaphylococcal antibiotic combined with a toxin inhibitor (clindamycin, linezolid or rifampicin), to reduce the use of excess antimicrobial treatment and surgical procedures (Kaushik and Kest, 2018; Urish and Cassat, 2020).

Although there are studies in Mexico that describe the characteristics of *S. aureus* in invasive infections, none have studied the clinical, microbiological and molecular characteristics of *S. aureus* in OAIs in pediatric patients (Garza-González et al., 2019; Vazquez-Rosas et al., 2021; Aguilar- Gómez et al., 2021). The aim of this study was to describe the phenotypic and genotypic characteristics of *S. aureus* isolates obtained from OAIs of paediatric patients, as well as their clinical characteristics, evolution and outcomes.

## Materials and methods

### Study site

This was a prospective unicenter study conducted at the National Institute of Paediatrics (INP), which is a tertiary care paediatric hospital located at the south of Mexico City. It is a referral, non- profit, teaching hospital with 251 beds and an annual average of 6,039 hospital admissions.

### Ethical aspects

This study was reviewed and approved by the research, ethics and biosafety committees of the INP (IRB: 00008064 and IRB: 00008065) under registration number 2019/007. Written informed consent was obtained from the parents or legal representative of the children, and informed assent was obtained from patients older than 12 years of age. The identity and data of the patients included were de-identified.

### Patient selection and sampling

We established a multidisciplinary surveillance program in our hospital called *OsteoCode* to identify patients with clinically suspected OAI. The patients were evaluated in clinical consultations by orthopaedists, radiologists, and infectologists. A collaborative approach was used to establish patient management, antibiotic and surgical treatments, and when necessary, samples (before, during or after surgery) were sent to the microbiology molecular laboratory, giving priority to standard care samples.

Children under 17 years of age were admitted to the INP from January 2019 to July 2021 with a diagnosis of OAIs were included. The OAI was considered when the patient had at least one of the following features: pain, limitation of movement, edema, erythema and/or fever plus an imaging studies (radiography, magnetic resonance imaging (MRI) and/or bone scintigraphy) compatible with these conditions (Woods et al., 2021). Joint fluid, exudate, tissue, biopsy or bone samples were taken from each patient during elective surgery as part of standard care. In all patients, blood cultures were obtained.

### *S. aureus* identification

The samples were cultured in brain-heart infusion (BHI), mannitol salt agar and trypticase soy agar (Becton Dickinson, Le Pont de Claix, France) and were incubated at 37°C for 18-24 h. Then, presumptive colonies were selected, and Gram staining and catalase tests were performed. DNA was obtained with the QIAamp^®^ DNA Mini Kit (QIAGEN, Hilden, Germany) following the manufacturer’s instructions. The DNA was eluted and stored at -20°C until use. The identification of *S. aureus* was performed by detection of the *nuc, sp*A and *femA* genes by PCR using previously reported primers (Brakstad et al., 1992; Relman, 1993). In all PCRs, AB9700 Thermocycler equipment (Applied Biosystems, Foster City, CA, USA) and AmpliTaq Gold^®^ 360 MasterMix (Applied Biosystems, Foster City CA, USA) were used. The 16S rRNA gene was also amplified, sequenced and analysed (3500 XL system, Applied Biosystems, Foster City, CA, USA) (Zhang et al., 2000; Morguilis et al., 2008). Additionally, *sp*A gene detection was performed through real-time PCR with previously reported primers and conditions (Okolie, 2017).

### Susceptibility profile

The susceptibility profile was performed following the Clinical Laboratory Standard Institute (CLSI) guidelines; the tested antibiotics were cefoxitin (FOX), clindamycin (CLI), gentamicin (GEN), erythromycin (ERI), trimethoprim-sulfamethoxazole (TMP/SMX), ciprofloxacin (CIP) and linezolid (LZD) (Becton Dickinson, Franklin Lakes, New Jersey, USA). The minimum inhibitory concentrations of vancomycin (VA) and teicoplanin (TEI) were determined with the broth microdilution method (CLSI, 2020).

The inducible resistance phenotype to macrolides, lincosamides and streptogramin B (MLSBi) was considered when isolates were resistant or intermediate to ERI, sensitive or intermediate to CLI and D-test positive; the MSB phenotype was considered when the D-test was negative; and the constitutive phenotype (MLSBc) was determined if they were resistant to ERI and CLI (Miklasińska-Majdanik, 2021).

### Resistance genes

In FOX-resistant isolates, the *mecA* gene was amplified (Louie et al., 2002), and the SCC*mec* element was determined by mPCR (Boye et al., 2007). The *ermA, ermB, ermC and msrA* genes were also detected (Khodabandeh et al., 2019; Fri et al., 2020).

### Biofilm formation

Biofilm formation was determined using the crystal violet method as previously described after culturing each isolate in BHI supplemented with 222.2 mM glucose, 116.9 mM sucrose, and 1000 mM NaCl. Optical density was measured at 550 nm. Isolates were categorized into non-biofilm former (NBF), weak biofilm former (WBF), moderate biofilm-former (MBF), and strong biofilm former (SBF) (Singh et al., 2017).

### Virulence factors

Eleven virulence genes were amplified by PCR, including adhesion proteins, fibronectin binding protein A (*fnbA*), fibronectin binding protein B (*fnbB*), clumping factor A (*clfA)*, clumping factor B (*clfB*), collagen adhesin (*cna*), invasion proteins: α-haemolysin (*hla*) and Panton Valentine leukocidin (*pvl*)*;* toxigenic, exfoliative toxin A (*eta*), exfoliative toxin B (*etb*), staphylococcal enterotoxin C (*sec*) and the super antigen toxic shock syndrome toxin 1 (*tsst-1*) (Azimian et al., 2012; Yeswanth et al., 2017).

### Molecular typing

We performed pulsed-field gel electrophoresis (PFGE) using the CHEF Mapper XA System (Bio-Rad, Hercules, California, USA) following the guidelines established in the protocol for MRSA Pulse Net of the Centers for Disease Control and Prevention (Centers for Diseases Control, 2002). The analysis was performed following Tenover’s criteria (Tenover et al., 1995). The ImageLab v6.1.0 program (Bio-Rad, Hercules, CA, USA) was used to create a 0/1 matrix, and DendroUPGMA (Garcia-Vallvé and Puigbo, 2022) and MEGA-X programs v.11 (Kumar et al., 2018). The sequence type (ST) of the isolates was determined by multilocus sequence typing (MLST) (Enright et al., 2000).

### Clinical data

An OAI by *S. aureus* was considered when the bacterium was isolated in at least one sample, either from joint secretion, joint fluid, tissue biopsy, bone samples or blood culture (Woods et al., 2021). OA can be defined as a condition characterized by focal areas of loss of articular cartilage within the synovial joints, associated with hypertrophy of the bone (osteophytes and subchondral bone sclerosis) and thickening of the capsule (Pereira et al., 2011).

The origin of OAIs was classified as haematogenous (primary bacteraemia), direct inoculation (trauma or surgery procedure) and contiguous (from infection adjacent to the skin and soft tissues) (Woods et al., 2021). In this study, OM was considered acute when the duration of symptoms was <14 days; subacute, >14 days and <3 months, and chronic >3 months (Alvares and Mimica, 2020).

Clinical information such as age, sex, comorbidity, risk factors, previous antibiotic treatment, duration of symptoms prior to diagnosis, clinical presentation, and type of infection were obtained at hospital admission. Patients were classified as neonates (0 days -27 days), infants (1 month- 12 months), toddlers (13 months-2 years), early childhood (2-5 years), middle childhood (6-11 years), and early adolescence (12-<18 years) (Williams et al., 2012).

Pain was categorized as mild, moderate, and severe using the visual analogue pain scale (VAS) (Crellin et al., 2021). Prolonged fever was defined when the patient’s temperature was 38°C during the four days following hospital admission (McNeil et al., 2019). For this study, the maximum fever peak was defined as the highest temperature level that the patient exhibited during their hospital stay.

### Laboratory and radiographic findings

The topography of the infection was described, and the following laboratory data were recorded on admission: white blood cell count, erythrocyte sedimentation rate (ESR) and C-reactive protein (CRP). All patients with OAIs had anteroposterior (AP), lateral and comparative extremity radiographs, and the presence of periosteal reaction was recorded in a radiolucent image to be separated from the cortical and lytic sclerosis for bone destruction. In patients without osteosynthesis material, an MRI was performed, and Brodie’s abscess was defined as evidence of an intraosseous abscess with a sclerotic wall and necrosis to the presence of a portion of free bone (sequestration) (Mandell et al., 2018; Urish and Cassat, 2020).

### Antimicrobial treatment

Previous treatment was considered to be the antibiotic administered during the six months before admission to the hospital; empirical treatment was defined as the antibiotic administered from the initial clinical evaluation until the microorganism and its susceptibility profile were identified; the antimicrobial targeted at the causal agent was recorded as definitive treatment, and outpatient treatment was indicated after the patient was discharged from the hospital. To standardize the duration of treatment in all cases, the day of microbiological sampling was considered Day 0.

### Surgical treatment, complications and sequelae

During the hospitalization of the patients, the number of surgical procedures, local treatment administered, complications and sequelae presented six months after the diagnosis of OAIs were recorded.

An orthopaedic complication was considered if the patient presented chronic OM, pathological fracture, physiatric injury (dysmetria or physiological arrest), avascular necrosis or dislocation (luxation). For this study, chronic complicated OM was defined if after four weeks of confirmed infectious process (Day 0) the patient presented at least one of the following: sequestration or lytic lesions in the bone visible by radiography, pain, erythema, edema, or loss of function of the affected extremity (McNeil et al., 2019).

Recurrent infection was defined as the presence of clinical data of infection with the same microorganism one month after the patient completed the treatment for the first episode, and recovery was defined as the clinical resolution of the infection after completion of antibiotic treatment without recurrence (Banerjee et al., 2020).

### Statistical analysis

IBM SPSS version 22.0 was used as the statistical program. Categorical variables were described as frequencies and percentages. Normality distribution was evaluated using the Shapiro Wilk´s method. Categorical variables were compared using Fisher’s x2 test. Normally and non-normally distributed data were compared with Student´s t-test and Mann- Whitney -Wilcoxon`s test respectively. Adjusted *p* < 0.05 were considered as significant.

## Results

Through *OsteoCode*, 80 patients were admitted to the hospital for suspected OAIs; in 67 cases, the diagnosis was confirmed, and in 40% (n=27) of them, *S. aureus* was identified as the causal agent.

### Identification and resistance profile

The *nuc, spA* and *femA* genes were detected in the 27 isolates. Twenty-five strains (92.6%) were MSSA, and their susceptibility profile was GEN 96% (n=24), CIP 92% (n=23), ERI 88% (n=22) and CLI 80% (n=20); all isolates were susceptible to TMP/SMX, LZD, VAN and TEI. The MLSB_i_ phenotype was detected in three isolates (O46, O61 and O86), and the MSB phenotype was detected in two (O48, O49). The two MRSA isolates were susceptible to TMP/SMX, LZD, VAN and TEI, and one of them (O35) was resistant to CIP, CLI and ERI (MLSB_c_ phenotype) (**Table 1**).

**Table 1:** Molecular characterization of *S. aureus* isolates.

Isolate	Resistance profile	MLSB profile (*ermA,ermC*, D-test)	Colonization (*clfA,clfB,fnbA,fnbB, cna*)	Invasion (*pvl, hla)*	Toxin/ SA (*eta, tsst-1*)	CC	ST	Biofilm
O1	None	None	*clfA,clfB*	*pvl*	None	45	152	MBF
**O2**	None	*ermA,ermC*	*clfA,clfB*	*hla*	None	5	5	NBF
O11	None	None	*clfA,clfB,fnbA,fnbB*	*pvl,hla*	None	ND	672	NBF
O13	None	None	*clfA,clfB,fnbA*	*hla*	None	5	ND	NBF
O14	None	None	*clfA,clfB,fnbA*	*hla*	None	5	6743	WBF
O19	None	None	*clfA,clfB,fnbA,fnbB*	None	None	ND	672	MBF
O23	None	None	*clfA,clfB,fnbA*	None	*eta*	5	6	NBF
O35	FOX*,CLI, ERI,CIP	*ermA*	*clfA,clfB*	*hla*	None	5	1011	NBF
O36	None	None	*clfA,clfB,can*	*hla*	None	ND	6561	MBF
**O38**	None	None	*clfA,fnbA*	*hla*	None	1221	ND	WBF
**O42**	None	None	*clfA,clfB,fnbB*	*hla*	None	8	72	MBF
O43	None	None	*clfA,clfB,fnbA*	*hla*	None	ND	672	MBF
O46	ERI, CLI^+^	*ermA*, D-test (+)	*clfA,clfB,fnbA,can*	*hla*	None	30	7222	WBF
O48	CLI, CIP	None	*clfA*	*hla*	None	8	8	NBF
O49	CLI, CIP	None	*clfA,clfB,fnbA*	None	None	1	9	NBF
**O52**	None	None	*fnbB*	None	None	15	15	NBF
O55	GEN	None	*clfA,clfB,fnbA,fnbB*	*pvl,hla*	None	ND	672	MBF
**O58**	None	None	*clfA,clfB,can*	*hla*	*tsst-1*	ND	707	SBF
**O59**	None	None	*clfA,clfB,fnbA*	*pvl,hla*	None	ND	672	MBF
O60	None	None	*clfB,can*	*hla*	None	1	188	NBF
O61	ERI, CLI^+^	*ermA*, D-test (+)	*clfA,clfB*	None	*tsst-1*	30	3994	NBF
O66	None	None	*clfB*	*pvl*	None	5	ND	WBF
O73	None	None	*clfA,clfB,can*	None	*tsst-1*	30	33	NBF
O74	None	None	*clfB*	None	*tsst-1*	30	30	WBF
O80	FOX**	None	*clfA,clfB,fnbA*	*hla*	None	ND	101	NBF
O86	ERI, CLI^+^	*ermA*, D-test (+)	*clfA,clfB,can*	None	*tsst-1*	30	1530	WBF
O94	None	None	*clfA,clfB*	None	None	8	8	MBF

FOX, cefoxitin; CLI, clindamycin; ERI, erythromycin; CIP, ciprofloxacin, GEN, gentamicin; *SCCmec-II; **SCCmec-V, +inducible MLS_B_ phenotype; MLSB, Macrolide-lincosamide-streptogramin B; erm, erythromycin ribosomal methylase,; +, positive; clfA, clumping factor A; clfB, clumping factor B; fnbA, fibronectin-binding protein A; fnbB, fibronectin-binding protein B; cna, collagen adhesin; pvl, Panton Valentine leukocidin; hla, α-haemolysin; eta, exfoliative toxin A; tsst-1, toxic shock syndrome toxin 1; CC, clonal complex; ND, no determinated; ST, sequence type; MBF, moderate biofilm-former; NBF, non-biofilm former; WBF, weak biofilm former; SBF, strong biofilm former. In bold, isolates not typed by pulsed-field gel electrophoresis. The genes ermB, msrA, sec and etb were not detected in any of the isolates.

### Resistance genes

MRSA isolates carried SCC*mec-*II (O35) and SCC*mec-*V (O80). The *ermA* gene was detected in 18.5% (n=5) of the strains; in one of them, coexistence with *ermC* (O2) was observed. Neither *ermB* nor *msrA* genes were identified (**Table 1**).

### Virulence factors

The *clfB* gene was the most frequent in 88.9% (n=24) of the isolates, and was followed by *clfA*, in 85.2% (n=23); *fnbA*, in 44.4% (n=12); *cna*, in 22.2% (n=6); and *fnbB*, in 18.5% (n=5). (**Table 1**).

The *hla* gene was detected in 59.3% (n=16) of isolates, and *pvl* was only observed in MSSA (18.5%, n=5). The *tsst-1* gene was amplified in 18.5% (n=5), and the *eta* gene was identified in one strain (O23). The *etb* and *sec* genes were not found (**Table 1**).

### Biofilm formation

Biofilm formation was detected in 55.6% (n=15) of the isolates; six of them were classified as WBF, eight MBF, and only one was SBF (O58) (**Table 1**).

### Molecular typing

**Figure 1:**
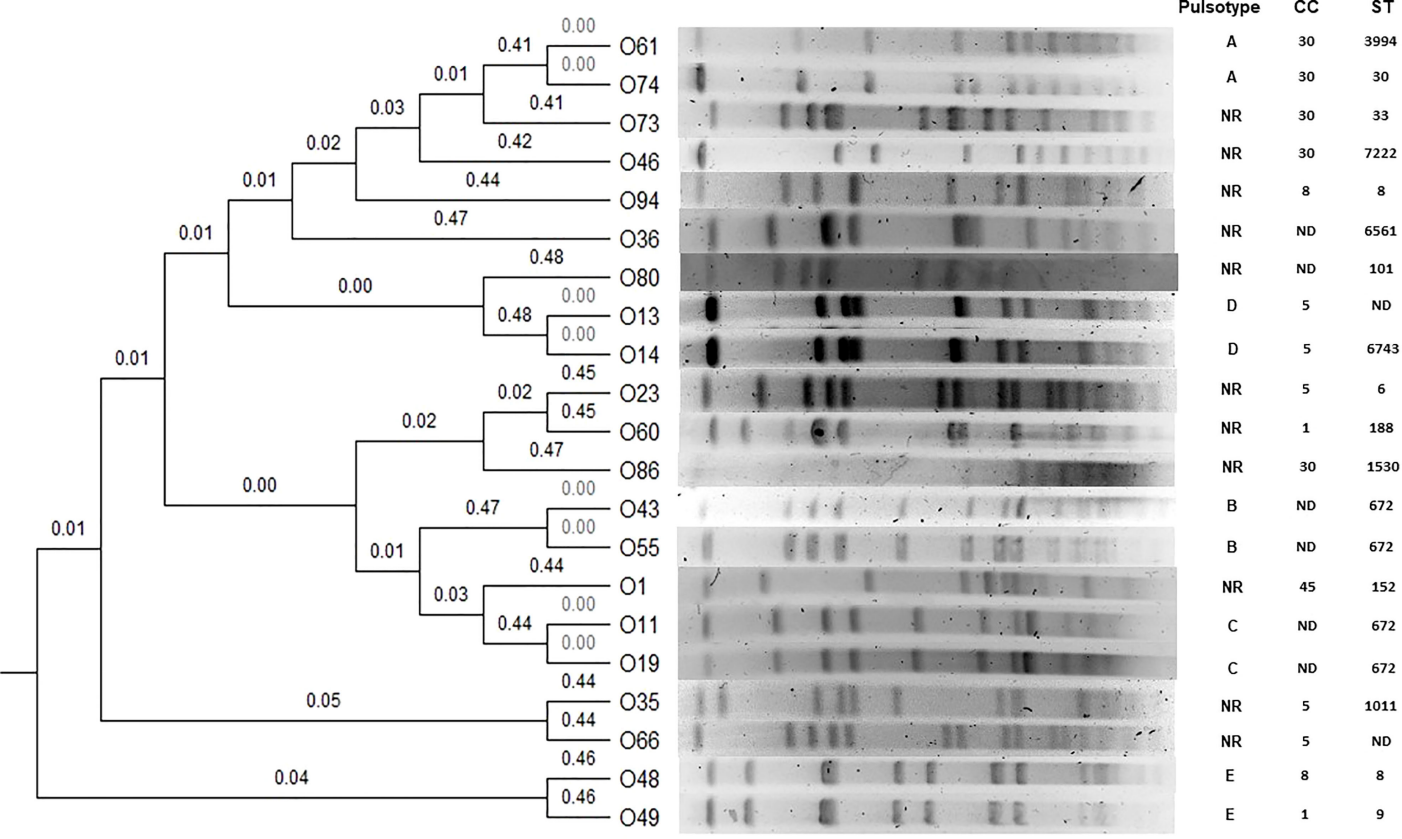
Dendrogram obtained by UPGMA (unweighted pair group method with arithmetic mean). Five clones are shown (A, B, C, D, E). NR, Non related; ND, Non determined.

Six isolates were nontypeable by PFGE (O2, O38, O42, O52, O58 and O59). Two isolates (O61, O74) were grouped into clone A, two (O43 and O55) into clone B, two (O11 and O19) into clone C, two (O13 and O14) into clone D, and two (O48 and O49) into clone E. Eleven isolates were considered unrelated (**Figure 1**, **Table 1**).

CCs had the following distribution among MSSA: CC5, 22.2% (n=6); CC30, 18.5% (n=5); CC8, 11.1% (n=3); CC1, 7.4% (n=2); CC15, 3.7% (n=1); CC45, 3.7% (n=1); CC1221, 4% (n=1), and in eight strains, the CC was not determined (O11, O19, O36, O43, O55, O58, O59 and CC80). Of the MRSA isolates, one belonged to CC5 (O35), and the second was not determined (O80). CC30 was associated with *tsst-1* (*p=*0.001).

MLST was performed on 24 isolates. The STs of MSSA were ST15, ST152, ST1530, ST188, ST30, ST33, ST3994, ST5, ST6, ST6561, ST6743, ST707, ST72, ST7222, ST9, ST8 (n=2) and ST672 (n=5), while the STs of MRSAs were ST1011 (O35) and ST101 (O80). For the rest of the isolates (n=3), the ST was not determined (**Table 1**). Several associations were observed: ST672 with *fnb*B and *fnbA* (*p=*0.003), *pvl* (*p*=0.030) and MBF (*p=*0.017).

### Clinical course, management, and outcome

OAIs were most frequent in adolescents (51.9%, n=14) andfifty-nine percent (n=16) of the patients were previously healthy. The patient diagnoses were OM (62.9%, n=17), SA (22.2%, n=6) and OA (14.8%, n=4). The most common origin of OAIs was haematogenous 55.6% (n=15). The onset of OM was acute (40.7%, n=11), followed by subacute (11.1%, n=3) and chronic (11.1%, n=3) (**Table 2**).

**Table 2:** Clinical, laboratory and outcome data of patients with osteoarticular infection due to *S. aureus*.

Isolate	Age	Sex	Comorbility	Infection	Origin of infection	Findings During Index Admission				LOS	Orthopaedic complication	Clinical outcome
						Fever (°C)	CRP (mg/dL)	ESR (mm/h)	Leukocytosis		
O1	Adolescence	M	None	OA	Haematogenous	**42**	4.2	72	N	47	COM, FP, LLD	Recurrence
O2	Early childhood	M	Genetic disorder	OM***	Haematogenous	No	0.3	2	N	18	None	Cure
O11	Middle childhood	M	None	OM*	Contiguous focus	**39.8**	0.4	50	Y	28	None	Cure
O13	Middle childhood	F	Congenital disorder	OM**	Contiguous focus	**38.2**	9.6	58	N	14	None	Cure
O14	Adolescence	M	Orthopaedic disorder	OM*	Direct inoculation	No	3	20	N	7	COM, LLD	Cure
O19	Middle childhood	M	None	SA	Haematogenous	**38.5**	33	53	Y	17	None	Cure
O23	Adolescence	F	None	OM***	Haematogenous	No	0.3	2	N	8	COM	Cure
O35	Adolescence	F	Genetic disorder	OM*	Haematogenous	**38.3**	11.3	50	N	41	None	Cure
O36	Adolescence	M	Oncological disorder	OM*	Contiguous focus	38	1	33	N	22	COM, LLD	Recurrence
O38	Adolescence	F	None	OM*	Direct inoculation	**38**	8.7	54	Y	16	FP, LLD	Cure
O42	Adolescence	F	None	OM**	Contiguous focus	No	0.1	40	N	16	None	Cure
O43	Early childhood	F	None	OM*	Contiguous focus	No	0	56	N	17	None	Cure
O46	Early childhood	F	Congenital disorder	SA	Haematogenous	39	2.5	62	N	6	None	Cure
O48	Adolescence	M	Genetic disorder	SA	Haematogenous	**38.1**	0	56	N	33	None	Cure
O49	Adolescence	M	Genetic disorder	OM***	Contiguous focus	**38**	16	53	N	27	COM, FP	Recurrence
O52	Adolescence	F	Genetic disorder	OM*	Direct inoculation	No	3.4	34	N	4	None	Cure
O55	Middle childhood	M	None	OA	Haematogenous	**39**	24	86	Y	46	COM, LLD	Cure
O58	Adolescence	M	None	OA	Haematogenous	**39.1**	17	64	N	30	COM	Recurrence
O59	Middle childhood	F	None	SA	Haematogenous	**38.7**	0.3	40	N	39	COM, FP, LLD	Recurrence
O60	Middle childhood	F	None	OM*	Haematogenous	**39**	4	66	N	8	None	Cure
O61	Middle childhood	M	None	SA	Haematogenous	No	1.6	6	N	6	None	Cure
O66	Adolescence	M	None	OM*	Haematogenous	No	0.7	47	Y	10	None	Cure
O73	Infancy	M	None	SA	Haematogenous	No	10	59	Y	39	None	Cure
O74	Infancy	M	None	OM*	Contiguous focus	No	0	10	N	39	None	Cure
O80	Adolescence	M	Oncological disorder	OA	Contiguous focus	**38.5**	3	70	N	50	None	Cure
O86	Middle childhood	F	None	OM**	Haematogenous	38	10	79	N	23	None	Cure
O94	Adolescence	M	Genetic disorder	OM*	Contiguous focus	38.3	3.6	35	N	22	None	Cure

M, male, F, female, OA, osteoarthritis, OM, osteomyelitis, SA, septic arthritis, *Acute, **Subacute, ***Chronic, CRP, C-reactive Protein, ESR, Erythrocyte sedimentation rate, Y, yes, N, no, LOS, length of hospital stays in days, COM, chronic osteomyelitis, PF, pathological fracture, LLD, limb length discrepancy. Isolates with pvl are underlined. Patients with prolonged fever are shown in bold.

History of trauma was documented in 40% (n=11) and was associated with *pvl* (*p*=0.047), *fnb*A and *fnb*B (*p=*0.041). Fever occurred in 63% (n=17), of which 76.4% (n=13) were prolonged. The isolates that were *pvl* positive presented a median fever of 39.4 °C (range 38.8-42) in comparison with negative *pvl* with 38.4 C (range 37.8-39.2) (*p=*0.015). Three cases (O1, O11 and O59) were initially evaluated by oncology for suspected osteosarcoma due to the magnitude of the bone lesion (**Table 2**).

According to the number of affected sites, the infection was considered multiple in 29.6% (n=8) of the patients. The most compromised joints in SA were the hip and knee in 11.1% (n=3, each), and in OM were the femur in 29.6% (n=8) and tibia in 25.9% (n=7) The presence of *pvl* was associated with multiple localization and soft tissue sites of infection beyond the bone (pyomyositis, pulmonary abscess) (*p=*0.017).

The median CRP level at admission was 3 mg/dL (range 0-33), while the median ESR was 53 mm3 (range 2-86). The patients with fever presented higher CRP median of 4.2 mg/dL (range 0.03-33) contrary to those without fever on admission with a median of 0.5 mg/dL (range 0.03-10) (*p*=0.013). ESR was more elevated in patients with *cna* carrying isolates with a median of 63 mm/h (range 33-79) different to negative *cna* with a median of 50 mm/h (range 2-86) (*p=*0.049). Leukocytosis was detected in eight patients, four of whom were diagnosed with OM (**Table 2**). The isolates with *fnbA* and *fnbB* had neutrophilia with a median of 15.5 10^9/L (range11.5-27.1) distinct from non *fnbA* and *fnbB* (median 6.1 10^9/L, range 3.0-21.0) (*p=*0.08). Both genes were also associated with leukocytosis (median 17.0 10^9/L, range 14.4-31.7) different from negative *fnbA* and *fnbB* (median 9.3 10^9/L, range 4.5-25.0) (*p=*0.08).

Nineteen patients received empirical antistaphylococcal antibiotic monotherapy (CFL); the rest received combined empiric therapy as follows: CFL+CLI (n=6); CFL+ cefotaxime (CTX) (n=1); and VA+CRO (n=1). Patients with MRSA received definitive treatment with VAN (O35) and LZD (O80); MRSA OAIs were associated with a longer duration of intravenous treatment and hospital stay median 45 days (range 41-50) (*p=*0.023) in comparation to MSSA isolates with a median of 18 days (range 4-47). The median duration of intravenous antibiotic therapy was 17 days (range 2-50). All outpatients received oral treatment; 55.5% (n=15) cefadroxil (CFR); 25.9% (n=7), CFL; 22.2% (n=4), CLI; and one, LZD (O80). The median duration of oral antibiotic therapy was 40 days (range 7-180) in outpatients (**Table 3**). In addition to antimicrobial treatment, surgical intervention was performed in 92.5% (n=25) of the patients, and in 37% (n=10) local treatment was administered, such as bioactive glass (60%, n=6) and calcium sulphate (40%, n=4).

**Table 3:** Antimicrobial treatment of patients with osteoarticular infection due to *S. aureus*.

Isolate	Infection		Empiric and definitive treatment (days)							Outpatient treatment (days)			
		RD	CFL	DC	CLI	VA	CRO	LZD	Total	CFL	CFR	CLI	Total
*O1*	OA	0	0	**1 to 43**	**1 to 43**				44		108		152
O2	OM	0	0	**1 to 16**					17		46		63
*O11*	OM	0	0, **1 to 27**		**1 to 27**				28	92			120
O13	OM	1	**1 to 11**			-1 to 0	-1 to 0		13	109			122
O14	OM	0	-1 to 0**, 1 to 8**						10		22		32
O19	SA	2	0 to 2, **3 to15**						16		12		28
O23	OM	1	0 to 1, **2 to 7**						8	173			181
O35	OM	4	-2 to 3			**4 to 37**			40		8+		48
O36	OM	3	0 to 3, **4 to 16**						17		67		84
O38	OM	1	0, **1 to 13**						14		15		29
O42	OM	1	0 to 1, **2 to 14**						15	15			30
O43	OM	1	-5 to 1, **2 to 11**						17			14	31
O46	SA	1	0 to 1, **2 to 5**						6			29	35
O48	SA	0	-1 to 0, **1 to 30**						31		24		55
O49	OM	0	-1 to 0, **1 to 24**						26		65		91
O52	OM	1	0 to 1, **2 to 14**						15		14		29
*O55*	OA	1	0 to 1, **2 to 44**		**2 to 44**				45		126	126	171
O58	OA	1	0 to 1, **2 to 34**		0 to 1				35			44	79
*O59*	SA	1	-11 to 1, **2 to 26**		-11 to 1, **2 to 26**				38	62			100
O60	OM	4	-1 to 4, **5 to 12**		-1 to 4				14			22	36
O61	SA	1	0 to 1, **2 to 5**						6	31			37
*O66*	OM	1	-1 to 1, **2 to 8**		**2 to 8**				10		20		30
O73	SA	2	0 to 2, **3 to 28**				0 to 2*		29		5		34
O74	OM	1	-4 to 1		**2 to 35**				40		36		76
O80	OA	1	-3 to 1		-3 to 1	**2 to 22**		**23 to 32**	36				67**
O86	OM	1	0 to 1, **2 to 20**		0 to 1, **2 to 20**				21		22		43
O94	OM	1	-7 to 1, **2 to 16**		-5 to 1				24	34			58

OA, osteoarthritis; OM, osteomyelitis, SA septic arthritis; RD, report day; CFL. cephalothin, DC, icloxacillin; CLI, clindamycin; VA, vancomycin; CRO, ceftriaxone; LZD, linezolid; CFR, cefadroxil. Definitive treatment is shown in bold. PVL isolates are indicated in italics, +patient underwent an amputation completing antimicrobial regimen for MRSA and was discharged with CFR to prevent skin and soft tissue infection after the surgical procedure, *patient treated with cefotaxime, **patient treated with oral for 31 days. In all cases, Day 0 was considered the day of sampling.

Orthopaedic complications occurred in nine patients (33.3%) and the most common was chronic OM (n=8). No patient developed sepsis or required admission to the paediatric intensive care unit. Among patients with complications, 83.3% (n=6) required hospital readmission and prolonged duration of hospital antimicrobial treatment (>40 days). Five children developed recurrent infection during the outpatient treatment. No significant difference in the orthopedic complications or recurrence and biofilm formation was observed (**Table 2**).

## Discussion

This is the first study in Mexico to describe MSSA as the most common causative agent of OAIs in the paediatric population. Only two isolates were MRSA, and one of them was a hospital-acquired (O35) infection with SCC*mec*-II that was also resistant to CLI, ERI and CIP. This finding is similar to that reported for MRSA, which presents resistance to fluoroquinolones (75-100%), GEN (59-100%), CLI (60-100%), ERI (71-100%) and TMP/SMX (30-97%) (Martínez-Aguilar et al., 2004; Davis and Gilbert, 2018). In the second MRSA isolate (O80), the SCC*mec*-V element was identified and has been mainly associated with community-acquired infections (Martínez-Aguilar et al., 2004; Davis and Gilbert, 2018).

MSSA is predominant in OAIs (58.3-98%) (Kechrid et al., 2011; Calvo et al., 2016; Bouras et al., 2018, Park et al., 2019), although a higher frequency of MRSA has also been reported in studies conducted in the USA, Argentina and Italy (45-100%) (Kini et al., 2013; Gaviria-Agudelo et al., 2015; Rosanova et al., 2015). In this study, 89% of the isolates were MSSA. In our hospital, dicloxacillin or first-generation cephalosporins (1GC) are both used as empirical treatment for OAIs, and this therapeutic strategy is due to the predominance of MSSA.

There is limited use of CLI in the empirical treatment of OAIs; however, it represents a therapeutic option due to its excellent bone penetration and toxin inhibition activity (PVL) (Alder et al., 2020). Resistance to CLI in this study was 20% among MSSA strains and was also observed in one MRSA strain; in another series, resistance ranged from 11.4% to 54% and from 8.1% to 44% in MRSA and MSSA, respectively (Kini et al., 2013; Bouras et al., 2018). The frequency of the *erm* and *msrA* genes has not been estimated in OAIs from paediatric patients, but it is recognized that their distribution varies according to the study region (Goudarzi et al., 2020; Miklasińska-Majdanik, 2021). The presence of these resistance mechanisms limits treatment with MLSB; therefore, efforts should be directed to the correct detection of constitutive or inducible resistance. All of the above reflects the importance of the empirical antimicrobial therapy of the *S. aureus* OAI according to the epidemiology of each hospital center.

In this study, the *clf*A gene was one of the main factors identified in MSSA strains (91.3%) and in the two MRSA strains; similar frequencies have been reported in other studies (100% in MSSA and 84.6% in MRSA) (Bouras et al., 2018).

Most *S. aureus* carry both *fnbA* and *fnbB*, and no differences in adherence have been observed when one or both are detected (Peacock et al., 2000). These factors are associated with invasive disease (Bocchini et al., 2006), increased virulence, IL-6 production, mortality, and weight loss (Palmqvist et al., 2005). In this series, the *fnbA* gene was detected in 44.4% of MSSA isolates, while *fnbB* was detected in 18.5%; similar to that reported in a study in which it was found in 50% of MSSA isolates (Bouras et al., 2018).

Cna protein has the capacity to bind to the bone matrix, whose composition is mainly collagen (90%) (Urish and Cassat, 2020). In this series, 24% of MSSA carried the *cna* gene; this result was similar to that found in another study, where it was present in 23.8% of MSSA strains (Bouras et al., 2018).

PVL toxin has been detected in both MSSA and MRSA isolates (Albiński et al., 2018; Hoppe et al., 2019). It is recognized that there are geographic differences in its distribution; in Europe, most *pvl*-containing strains are MSSA (Bouras et al., 2018; Hardy et al., 2019), while in the USA, they are predominant in CA-MRSAs (74-100%) (Ritz and Curtis, 2012). In Mexico, PVL toxin detection is not routinely performed, so there is underreporting; it has been observed that patients infected with PVL-producing strains have longer fever, multiple localization, extraosseous complications such as pyomyositis, septic emboli, chronic OM, and elevated inflammatory markers (Davis and Gilbert, 2018). In this study, *pvl* was detected in 18.5% of the strains, all were MSSA, and their presence was associated with high fever, multiple localization and pyomyositis. It is important to consider the routine detection of PVL to determine its frequency and establish its relationship with symptomatology and severity in invasive diseases caused by *S. aureus*.

The *tsst-*1 gene was detected in 18.5% of isolates; a similar frequency (17.9%) was reported in a study of 84 isolates, and patients were observed to have an early stage of infection and elevated ESR (Kim et al., 2015). In our series, *tsst*-1 was more frequent in strains from patients with SA (37.5%) than in those from OM (15.8%). The presence of superantigens in OAIs has been described mainly in those associated with prosthetic devices (92.9%) (Kim et al., 2015).

Biofilm formation is one of the most important aspects in the development of *S. aureus* infections, such as endocarditis, OM, and SA, and is associated with the use of medical devices (Cue et al., 2012). In our study, 55.6% of the isolates were biofilm producers; only one was SBF, which carried *clfA, clfB, hla, cna* and *tsst-1*. No MRSA isolates were biofilm-forming. One study showed that patients with OM had a reduced inflammatory response if they were infected with *S. aureus* SBF and that there were no significant differences in biofilm production or antimicrobial resistance between OM and non-OM isolates (*p=* 0.946) (Yu et al., 2020); in our study, we found no differences between producers and non-producers with clinical, laboratory, or molecular characteristics of the strains.

Information about the clonal distribution of *S. aureus* causing OAIs in the paediatric population is limited (Pimentel de Araujo et al., 2021). In a study in which 68 isolates were analyzed, 92.3% of MRSA showed the same pulse type, whereas in MSSA, they had different clonal origins (Bouras et al., 2018). In this collection, this phenomenon was also observed among MSSA; however, five clones (A, B, C, D and E) could be distinguished.

In our study, CC5 followed by CC30 and CC8 were the main CC detected which coincides with the isolates reported in the epidemiology of *S. aureus* infections, in Mexico (Vazquez-Rosas et al., 2021). Also, this is consistent with other authors who point out the diversity of origins of strains causing paediatric OAIs, particularly in MSSA (Kechrid et al., 2011; Yu et al., 2020; Pimentel de Araujo et al., 2022).

In this series, in three isolates the STs were not determined, but the MLST analysis allowed us classified them into CC5 (O13 and O66) and CC1221 (O38). We obtained a large diversity of STs in the MSSA isolates however, ST672 and ST8 are the most prevalent; this is contrasting to other studies which informed that ST30 is predominant among MSSA (Park et al., 2019). To our knowledge ST672 has not been reported in our country till now. According to the MLST database, 20 isolates of ST672 have been recorded since 2003 in India, USA, Australia, Iran and Haiti (Jolley et al., 2018); This clone has participated in invasive diseases such as septicaemia, urinary tract infection and pneumonia, and all of them harbored SCC*mec* type V (Jolley et al., 2018). In non-invasive diseases like pharyngitis and skin infection the isolates were MSSA (Jolley et al., 2018). All previous exhibits the possible community origin of this clone.

Interestingly in this study, ST672 was associated with *fnb*B and *fnb*A (*p*=0.003) and *pvl* (*p*=0.030) which are virulence factors related to invasive disease (Palmqvist et al., 2005; Davis and Gilbert, 2018). In 2012, ST672 was reported as an emerging clone in India. The clinical isolates were MRSA SCC*mec* type V and the carrier isolates were MSSA, both without PVL (Shambat et al., 2012; Khedkar et al., 2012). In Australia, Coombs et al. reported ST672 carrying SCC*mec*-IV and V (Coombs et al., 2011). In Haiti, in one study from 10 *S. aureus* clinical isolates (skin and soft tissues infections and empyema) ST672 was identified in one isolate. This strain was categorized as CC672 and was from MSSA penicillin susceptible and PVL- positive (Schaumburg et al., 2015).

In this series the ST of the two MRSA isolates were ST1011 and ST101 different from other studies that demonstrated ST8, ST80, ST728 (CC8) and ST30 (CC30) as the most common among MRSA isolates from paediatric patients with OAIs (Gaviria-Agudelo et al., 2015; Bouras et al., 2018).

Although no epidemiological factor was found that could relate the patients and explain the PFGE results, it is important to note that isolates from clone A belonged to CC30, clustered in distinct STs and carried the *tsst*-1 superantigen gene, while those from clone D clustered in CC5 and had a similar virulence profile. Isolates from clones B (ST672) and C (ST672) and showed different virulence profiles. The isolates from clones 5 belonged to different CC and STs with distinct virulence profile.

Fever is a late clinical sign, and its absence does not exclude the diagnosis of OAIs. In a retrospective study, fever for more than 48 h was observed to be a predictor of acute complications in haematogenous OM in pediatric patients (Alhinai et al., 2020). In this study, fever occurred more frequently in SA cases than in OM cases (71.4% and 38.4%, respectively). The magnitude of fever has been associated with MRSA (Davis and Gilbert, 2018). In our study, peak fever was associated with the presence of *pvl*, and in the literature, it has been shown that the expression of this factor is related to higher virulence and severity (Calvo et al., 2016; Davis and Gilbert, 2018).

In one study, patients with OAIs due to MRSA in the early stages of the illness were reported to have elevated inflammatory markers, such as CRP, ESR, fever and total neutrophil count. Elevated CRP (≥100 mg/L) after 2-4 days on antimicrobial therapy predicts an increased risk of morbidity (OR 2.7 95% CI (1.0-7.3) (Alhinai et al., 2020). In this series, leukocytosis was associated with f*nb*A and *fnb*B genes in *S. aureus* patients (*p=*0.08), while elevated ESR was related to *cna*.

Currently, the treatment of OAIs is a challenge and includes antimicrobial therapy with control of infectious foci. The antibiotic choice should consider several factors, including age, drug toxicity, bone penetration, and local prevalence of methicillin-resistant *S. aureus*. ln this study, according to current recommendations the empiric therapy was with first-generation cephalosporins (1GC) or dicloxacillin (DC), since in our hospital we have low rates of Community- associated MRSA infections (CA-MRSA) (less than ~10%) (Saavedra-Lozano et al., 2015; Castellazzi et al., 2016; Bréhin et al., 2020; Woods et al., 2021; Krzysztofiak et al., 2021). In seven children, we added to the empiric therapy with DC or 1GC a protein synthesis- inhibiting antibiotic as clindamycin. We suspected a toxin- mediated disease due to that they presented OM with high or prolonged fever, multiple localizations, lesion in soft tissue sites of infection beyond the bone. Actually, there is no data that compares treatment regimens for children with known toxin-mediated staphylococcal infections and the presence and role of toxin production in disease severity remain uncertain (Woods et al., 2021). On addition there is no consensus about the treatment duration in serious and complicated infections. In this study, the treatment duration was individualized and we considered discontinuation after a multidisciplinary assessment that evaluated an adequate focus control, clinical and paraclinical improvement.

Complications in patients with OAIs due to *S. aureus* have been reported in 9.4%, although the origin has been described to be multifactorial; prolonged fever, *agr III* group and delayed control of the infectious process have been associated with its presentation (McNeil et al., 2019). Particularly in paediatric patients with haematogenous OM, acute and chronic complications are more frequent, occurring in 24% and 11%, respectively (Alhinai et al., 2020). In this study, 33.3% of patients developed orthopaedic complications. Different studies have shown that OAIs due to MRSA are more virulent and invasive and are associated with more surgical procedures, complications, longer length of hospital stay, higher costs, and mortality than those caused by MSSA (Bouras et al., 2018; Davis and Gilbert, 2018). Patients with MSSA had a higher number of surgical procedures and presented more complications, which could be explained by the detection of different virulence factors that are involved in the pathogenesis of the infection and severity. In this study, the two MRSA strains were associated with a longer duration of treatment and length of hospital stay, but no differences were found in the number of surgical interventions, complications, or mortality.

An important limitation of this study was the sample size. However, it allowed us to know the characteristics of *S. aureus* as a causal agent of OAIs in a tertiary care hospital through the implementation of the *OsteoCode*. The main objective of this initiative was to form a team of orthopaedists, radiologists, infectologists, microbiologists and rehabilitators with the propose to make a timely diagnosis, adequate treatment and follow-up until the resolution and recovery of the OAIs. Additionally, the epidemiological and molecular characteristics of the strains were studied.

Given the complexity of detecting these infections, our results provide the basis for future multicenter studies systematizing a multidisciplinary approach for the detection, management, and follow-up of these pathologies.

To our knowledge, this is the first study that informs the epidemiology of *S. aureus* in OAIs in paediatric patients in Mexico; a clear predominance of MSSA distributed in different CCs was observed. The severity of the cases can be explained by the production of different virulence factors rather than by methicillin resistance. The adhesion factor *clf*B was the main factor; however, other strategies are required to determine its role in OAIs. PVL toxin was detected only in MSSA and was associated with multiple localization, pyomyositis and high fever. In children, OAIs are neglected pathologies and mainly affect patients without comorbidities. Our findings highlight that a multidisciplinary team is required for successful diagnosis and treatment of OAIs.

## Data availability statement

The data presented in the study are deposited in the GenBank, accession number OP765299-OP765319 and OP795510-OP795657.

## Ethics statement

The studies involving human participants were reviewed and approved by research, ethics and biosafety committees of the Instituto Nacional de Pediatria (IRB: 00008064 and IRB: 00008065). Written informed consent to participate in this study was provided by the participants’ legal guardian/next of kin.

## Author contributions

NA-G and AA-A contributed to conception and design of the study. NA-G, EJ-B, JM-V, and AA-A performance experiments. OI-A, MM-P, OC-M, and NA-G enrolled patients and collected clinical data. NA-G and SG-P performed the statistical analysis, NA-G and AA-A wrote the first draft of the manuscript. AA-A acquired funding. All authors contributed to manuscript revision, read, and approved the submitted version.

## Funding

This study was supported by modality A of fiscal resources for 2019, 2020 and 2021 of Instituto Nacional de Pediatria under registration INP-2019/007 and by the Young Investigator Awards 2019 by Institut Mérieux to AA-A.

## Acknowledgments

We thank Dr. Carolina Romo Gonzalez and Dr. Rafael Franco Cendejas for the technical support with the biofilm formation technique.

## Conflict of interest

The authors declare that the research was conducted in the absence of any commercial or financial relationships that could be construed as a potential conflict of interest.

## Publisher’s note

All claims expressed in this article are solely those of the authors and do not necessarily represent those of their affiliated organizations, or those of the publisher, the editors and the reviewers. Any product that may be evaluated in this article, or claim that may be made by its manufacturer, is not guaranteed or endorsed by the publisher.
